# How the Registration of Nurses Would Benefit the Nurses and Physicians, as Well as the Public

**Published:** 1907-08

**Authors:** A. C. Hartridge

**Affiliations:** President Association of Nurses of Georgia, Atlanta, Ga.


### *HOW THE REGISTRATION OF NURSES WOULD BENEFIT THE NURSES AND PHYSICIANS, AS WELL AS THE PUBLIC. *

MRS. A. C. HARTRIDGE,
President Association of Nurses of Georgia, Atlanta.

The modern movement for legal status of the Nursing profession had its beginning in 1889, when the Royal British Nurses Association was founded for the purpose of securing a system of registration. Although fully endorsed by the British Medical Association, the movement met with such bitter opposition that only within the last three years, by special Act of Parliament, a Central Nursing Board was created for the purpose of defining the minimum educational standard of training schools.

That the movement has become general will be recognized from the fact that registration for Nurses now exists in South Africa, New Zealand, Australia, Germany and in sixteen of our own States. An examination of the legislation secured in these States will show that no new departure has been attempted; only the same protection for the public and the profession has been sought in the case of trained nurses as that which the profession of law, medicine and various other callings have long enjoyed. Only a little over fifty years ago Florence Nightingale demonstrated to the world that the application of theoretical training to the manual trade, would raise nursing from a menial service to a profession. Today this same profession has schools and teachers, tuition fees and scholarships, systems of instruction from preparatory work to post-graduate courses; is allied with technical schools on one hand and universities on the other. It has libraries, literature and a fast growing number of periodicals owned, edited and published by nurses. It has societies and laws, and has now arrived at a point when legal protection seems a vital necessity, not only for the benefits that will extend to its members, but for the benefits that will result in a larger measure to the medical profession and in a still larger measure to the general public.

*Read before the Fifth District Medical Association at Douglasville, Ga., July 17, 1907.

Twenty years ago there were few training schools in this country. Nursing was done by orderlies, ignorant women occupying menial positions, and frequently by convalescent patients in the free wards. With such assistance, modern surgery as we see it practical today in the operating rooms of our large hospitals, was an impossibillity.

In 1903 the United States Bureau of Education gave the total number of training schools for nurses as 552, and estimated that the total number of untrained women practicing as nurses was nearly three times as great as the number of regularly qualified nurses. With such rapid progress, it is not to be wondered that many important features relating to the welfare of both the pupil and the public were overlooked. If all training schools could have been qualified to turn out properly trained nurses—if they could have a fixed educational standard, the correspondence school would not have come into existence, and the dismissed probationer, masquerading in a graduate nurses uniform, would have found it much more difficult to impose upon the public. Registration as a means of purifying the profession would have been unnecessary, for all graduate nurses would have had the same educational qualifications; but with no recognized responsibility on the part of the training schools or the medical profession, or of the nurses themselves, to maintain a fixed standard, conditions have grown more and more chaotic, and the need for legislation must now be patent to all.

In the State of Pennsylvania with a Graduate Nurses Association numbering two thousand members, many of whom have given three years to the acquirement of their profession, a bill supported by the Association, calling for a state examining board for nurses has been defeated year after year. The reason is obvious.. The Philadelphia Correspondence School for Nurses publishes and scatters broadcast throughout the country bulletins which promise a complete course of instruction in ten weeks, without residence or any connection with any hospital. Its graduates are assured of $25.00 per week salary, are furnished with attractive looking diplomas and are turned out by the hundreds. In Camden, New Jersey, a nursing course of eight months is offered for the reasonable sum of $50.-

00, also without any connection with any hospital. In New York the Chautauqua School of Nursing, incorporated under the laws of the State, furnishes still another type. As both New York and New Jersey are protected by registration, Pennsylvania has thus become a haven of the correspondence school graduate, the number being so overwhelmingly strong as to yearly defeat all attempts at legislation.

The demands of modern medicine and surgery require that the nurse of today be something more than a mechanical machine to carry out orders. While her ability to carry out her orders absolutely and to the smallest detail is one of the tests of her professional fitness, at the same time it is also required of her that she intelligently understands these same orders, knows the reason for them and when to anticipate them. Refinement, education and prescribed professional training should be demanded of the graduate nurse who is to have charge of your training schools, the care of your patients and a definite standing in public life. That there are other qualifications no less important, such as ability to give loving service, sympathy, unselfishness, tact and a keen sense of honor, we all well know; but is it not more reasonable to suppose that these latter virtures will be more often found hand in hand with the former than in the company of ignorance and general incompetency? In no other professions are the sins and short-comings of the individual so visited upon the whole. Cases have been frequently cited here in our own State of gross breaches of professional etiquette and of criminal negligence and ignorance on the type of woman which the great nursing body condemns and deplores. Therefore, we are asking that a distinctive title other than graduate nurse be given legally to the nurse who is professionally grounded and ethical, in order to publicly distinguish her from the ignorant, unprofessional and generally untrustworthy nurse. "As a body we are contending that the more highly cultivated the woman and the better trained the nurse, the more willingly she serves the doctor, never infringing upon his province in the treatment of the patient, and using her medical knowledge independently only in cases of extreme emergency and at such times and in such manner as she knows would meet with his approval."

Thus, the American Journal states in an editorial commenting on certain changes to the effect that the nurse of the day is being over-trained and that the tendency of the profession is to infringe upon the doctor's prerogatives. Your own Interstate Medical Journal for April in referring to the same charges, made with the hopes of thus defeating registration in a certain state, says:

"Concerning insubordination on the part of nurses, any clear-sighted physician need not ask himself once (if he greatly values his medicines) to be convinced of which sort of a nurse in all human probability would be more likely to cast his decoctions out of the window—would it be the educated, refined woman, who understands rational therepeutics, or would it be the Sairy Gamp, who has long administered Catnip tea and done obstetrics on her own responsibility?"

State registration is still in its infancy, yet on every side we have improved conditions in consequence. It is not only setting and maintaining a standard of excellence and nursing education, but is furnishing an incentive to keep all nurses up to that standard. It is marking a step in the advancement of medicine by furnishing the practitioner with more intelligent and more reliable service. It is proving to nursing schools the necessity for a more uniform curriculum, and above all, it is enabling the public to determine by the only feasible means, whom it may safely apply to for skilled nursing in time of need. It has proved that it in no way menaces the liberty and usefulness of the nurse who cannot meet its requirements (unless it is that by force of contrast, when the educated nurse is recognized as being on a higher plane, the uneducated may seem to be lower than before in consequence,) for all enactments and proposed laws plainly state that in no way shall they be so construed as to interfere with any one practicing nursing, either for hire or for charity or in her own family, provided she does not assume the title of Registered Nurse. Registration will not make it harder for the qualified nurse to follow her profession than it will make it easier for the unqualified. Neither will it insure that good nurses are always good women. It will not deprive the public of its rights to make a free choice by compelling it to employ only Registered Nurses, but it will guarantee to it and to the medical profession that a nurse bearing the title of "R. N." has been qualified for the sick by careful and thorough trained in each of the general branches of her work, and that she is in good moral and professional standing in her training school and in the community in which she lives.

